# Online Intervention on Lung Cancer Screening Among High-Risk Individuals: Pilot Intervention Study

**DOI:** 10.2196/89823

**Published:** 2026-06-16

**Authors:** Fang Lei, Feifei Huang, Yufang Guo, Sun S Kim, Yang Liu

**Affiliations:** 1 School of Nursing, University of Minnesota, 308 Harvard St SE, Minneapolis, MN, 55455, United States, 1 612-626-7021; 2 School of Nursing, Fujian Medical University, Fuzhou, Fujian, China; 3 School of Nursing and Rehabilitation, Shandong University, Jinan, Shandong, China; 4 Department of Nursing, Donna M. and Robert J. Manning College of Nursing & Health Sciences, University of Massachusetts Boston, Boston, MA, United States; 5 Department of Computer Information Systems, School of Business, North Carolina Central University, NC, NC, United States

**Keywords:** education, intervention, lung cancer screening, online, smoking, artificial intelligence, AI

## Abstract

**Background:**

Lung cancer remains the leading cause of cancer deaths in the United States; however, uptake of lung cancer screening (LCS) with low-dose computed tomography (LDCT) among eligible individuals remains low. Evidence suggests that limited knowledge, stigma, and false health beliefs contribute to the underuse of LDCT screening.

**Objective:**

This pilot study aimed to examine an online educational intervention designed to improve knowledge, attitudes, health beliefs, behavioral intentions, perceived importance, and confidence related to LCS among high-risk individuals.

**Methods:**

A single-group preintervention and postintervention design was used. High-risk individuals who smoke, defined according to the US Preventive Services Task Force criteria, completed baseline questionnaires followed by 5 self-directed online educational modules delivered through Research Electronic Data Capture (REDCap). Postintervention questionnaires assessed changes in lung cancer and screening knowledge, lung cancer stigma, health beliefs based on the health belief model and precaution adoption process model, and intentions, perceived importance, and confidence regarding LDCT screening. LCS uptake was assessed via follow-up email 3 months after the intervention. Data were analyzed using descriptive statistics and paired-samples two-tailed *t* tests.

**Results:**

A total of 25 participants completed the intervention. Significant improvements were observed across all major study outcomes. Knowledge scores increased markedly (score=3.76-8.60; *P*<.001), while lung cancer stigma decreased (score=25.52-19.16; *P*<.001). Health belief model constructs showed significant improvements, including perceived susceptibility, perceived benefits, cues to action, and self-efficacy, alongside reductions in perceived barriers and perceived severity (all *P*<.001). Self-reported intentions, perceived importance, and confidence related to obtaining LDCT screening increased significantly. Of the 22 (88%) participants who completed the 3-month follow-up, 13 (59.1%) reported obtaining LDCT screening. Participant satisfaction with the intervention was high, with a mean score of 18.32 (SD 2.33) out of 20.

**Conclusions:**

Findings from this pilot study support the feasibility, acceptability, and preliminary efficacy of an online educational intervention created to promote LCS among high-risk individuals. The intervention improved knowledge; reduced stigma; positively influenced health beliefs; and increased screening intentions, perceived importance, confidence, and uptake. Results provide a foundation for a larger-scale study and suggest that online educational platforms may be an effective strategy to reach geographically diverse high-risk populations and promote LDCT screening.

## Introduction

### Lung Cancer Incidence, Mortality, and Survival Rates

Lung cancer is the leading cause of cancer deaths and the second most commonly diagnosed cancer in both male and female individuals in the United States [1]. In 2026, it is estimated that 229,410 people will be diagnosed with lung cancer, and 124,990 people will die from lung cancer [1]. Patients with lung cancer have one of the lowest 5-year survival rates (18.6%) compared to other types of cancers in the United States, such as colorectal (64.5%), breast (89.6%), and prostate (98.2%) cancers [2]. Only 16% of lung cancers are diagnosed at a localized stage, for which the 5-year survival rate is 55%. As the length of delay before diagnosis increases, the 5-year survival rate for patients with lung cancer significantly decreases. For example, when lung cancer is diagnosed at an advanced stage (stage IV), the survival rate drops to 4%. Additionally, more than half of people with lung cancer die within 1 year of being diagnosed [2].

### Primary and Secondary Preventions for Lung Cancer

Tobacco use is the most important risk factor of lung cancer, which contributes to 80% of lung cancer deaths in the United States [3]. Other risk factors of lung cancer include exposure to radon gas, occupational or environmental exposure to secondhand smoke, asbestos, certain metals, some organic chemicals, radiation, air pollution, and diesel exhaust. The primary approach to prevent lung cancer is smoking cessation, which has been proven to effectively decrease the incidence rates of lung cancer among male and female individuals [3].

Lung cancer screening (LCS) with low-dose computed tomography (LDCT) is an effective secondary prevention method [4]. It has been shown to reduce the mortality of lung cancer by 20%, compared to the standard chest x-ray, among high-risk individuals [4]. Screening for individuals at high risk for lung cancer (individuals aged between 50 and 80 years, who have smoked at least 20 pack-years, and currently smoking or having quit smoking within the past 15 years) [5] has the potential to improve lung cancer survival rates by detecting nodules at an earlier stage when it is more likely to be curable. It is reported that about 8 million Americans qualify as high risk for lung cancer and are recommended to receive annual screening with LDCT scans [5]. If half of these high-risk individuals are screened, more than 12,000 lung cancer deaths could be prevented annually [6].

### Guidelines and Uptake Rate of LCS

Since 2013, the US Preventive Services Task Force and other organizations (eg, American Cancer Society, American College of Chest Physicians, American Society of Clinical Oncology, and American Lung Association) have issued guidelines for early detection of lung cancer with yearly LDCT among high-risk populations [7]. It was covered both by the private and public health insurances for the high-risk population [8].

Although the supportive landscape for screening lung cancer among high-risk populations has improved, uptake rates of LCS with LDCT remain low [9]. The percentage of the eligible population who had received LCS with LDCT increased from 3.3% in 2010 to 3.9% in 2015 and reached 18.2% in 2022 among the US population [10,11].

### Barriers to LCS Uptake

Barriers to LCS for eligible individuals have been reported to include a lack of knowledge, practical challenges, financial constraints, psychological obstacles such as worry and anxiety, blame and stigma, fear of cancer, and fatalism [12]. Additionally, there is confusion surrounding LCS and a general distrust of the medical system. High-risk individuals seem to have limited knowledge about LCS. This lack of knowledge may stem from the little information they received regarding the screening process. Although LCS with LDCT is covered by both private and public health insurance for high-risk populations, awareness of this coverage appears insufficient, given the financial barriers frequently reported (eg, cost of the screening, insufficient authorization of health insurance reimbursement by insurance companies, and misunderstanding-associated out-of-pocket cost). In addition, stigma and fatalism toward the disease (eg, the perception that lung cancer is not treatable) could also impede high-risk individuals from screening for lung cancer [12].

### Interventions to Increase LCS Uptake and Limitations

Multiple interventions have been implemented to increase LCS uptake in high-risk populations. Evidence-based strategies include shared decision-making tools and decision aids designed to support informed screening choices and improve adherence to LCS guidelines [13]. In addition, a range of patient-directed interventions have been evaluated, including educational videos and information films [14] and clinical outreach and referrals [15]. However, prior studies vary substantially in design, intensity, and delivery context, with many interventions implemented within clinical settings or as part of health care system–based programs. Furthermore, most reports in this area are limited by focus primarily on describing barriers rather than evaluating intervention effectiveness. These limitations highlight the need for rigorously designed, scalable interventions that can be delivered outside of clinical environments and evaluated using standardized outcome measures.

In recent years, several rigorously developed digital interventions have been designed to support LCS decision-making. LungTalk, developed by Carter-Harris et al [16], is a theory-driven, web-based intervention that addresses lung cancer stigma, knowledge, and health beliefs to promote informed screening decisions. Similarly, mPATH-Lung (mobile Patient Technology for Health-Lung), developed by Miller et al [17], is a personalized, web-based decision aid that has been implemented in clinical settings to facilitate shared decision-making for LCS. In addition, LCS decision support tools developed by Volk et al [18] at MD Anderson Cancer Center provide evidence-based, patient-centered resources to guide screening decisions. These interventions demonstrate that digital approaches to LCS are both feasible and effective. However, despite these advances, many existing digital interventions are designed primarily as decision aids within clinical workflows or target-specific populations (eg, current smokers engaged in health care systems). In contrast, fewer interventions have focused on scalable, fully remote, multimodule educational programs that can be delivered outside of clinical encounters and are tailored to high-risk populations with limited access to LCS resources.

This study addresses the gap by developing and pilot-testing a structured, 5-module digital intervention integrating educational content, health belief modification, and culturally relevant messaging. Unlike existing intervention programs that primarily focus on facilitating a single screening decision, this study emphasizes a broader continuum of behavior change, including knowledge enhancement, belief modification, and intention formation. Additionally, the asynchronous and low-cost delivery format enhances scalability and accessibility, particularly for populations who may face barriers to clinic-based decision-making tools.

### Aims

This study aims to (1) test the feasibility of an online intervention program on increasing LCS uptakes among high-risk individuals and (2) assess the intervention’s effects on changing participants’ knowledge of lung cancer and LCS, attitudes toward lung cancer stigma, health beliefs regarding LCS, behavioral intentions, perceived importance, and confidence in obtaining LCS when compared to baseline measurements.

### Significance

This study implemented an online intervention aimed at increasing LCS uptake among high-risk individuals. Promoting LCS projects helps high-risk individuals better understand their risk of lung cancer, acquire knowledge about lung cancer and screening procedures, and enhance their awareness of the importance of LCS.

## Methods

### Design

**Figure 1.**
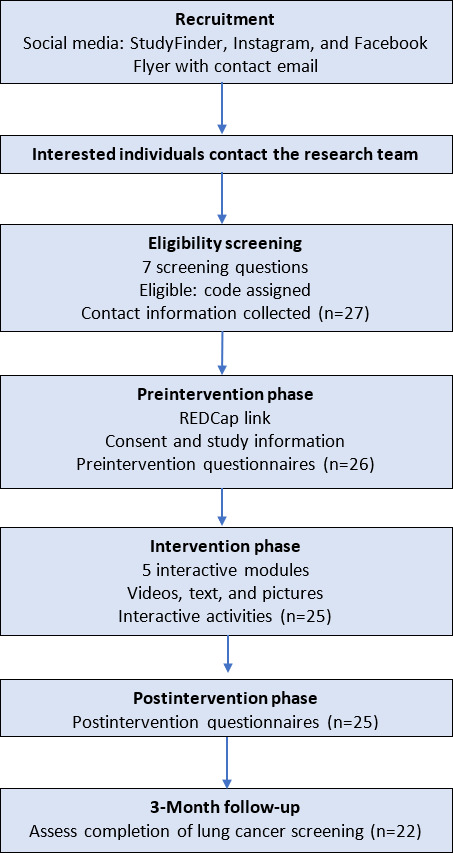
Study flowchart.

A single-arm preintervention and postintervention study design was used in this study. A flow diagram about the study process is shown in Figure 1. Paired-comparison data before and after the intervention were obtained and analyzed.

### Theoretical Framework

**Figure 2.**
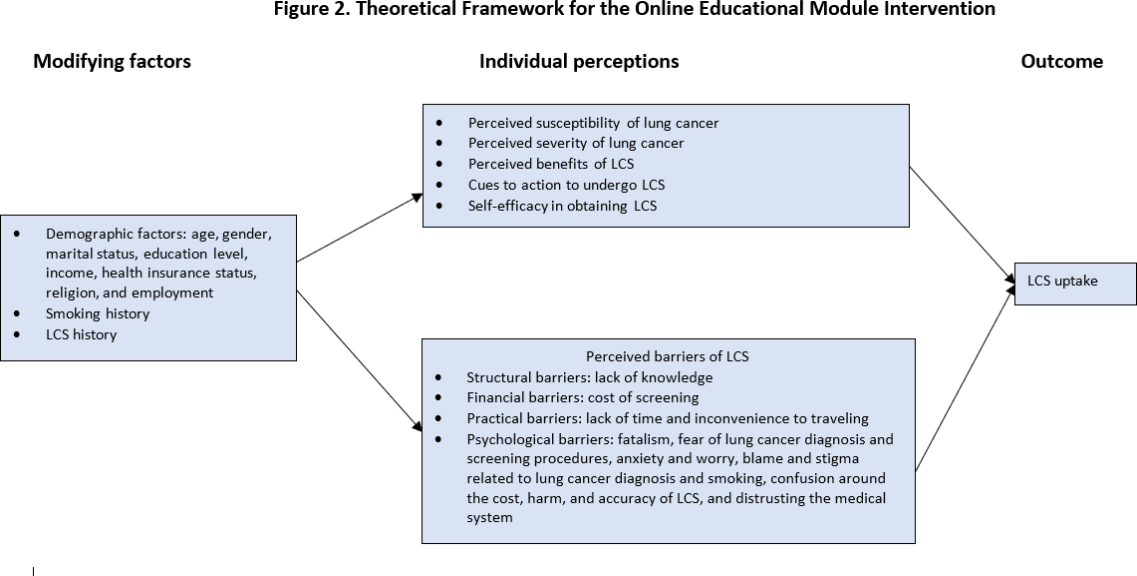
Theoretical framework for the online educational module intervention. LCS: lung cancer screening.

The health belief model (HBM) provided a theoretical framework for the study (Figure 2) [19]. Key concepts of the HBM are perceived susceptibility, perceived severity, perceived benefits, perceived barriers, self-efficacy, and cues to action [19]. In addition, this study was conceptually informed by the integrated LCS participation model developed by Carter-Harris et al [20], which incorporates HBM constructs alongside lung cancer stigma and the precaution adoption process model (PAPM). This integrated framework emphasizes that LCS participation is not solely driven by health beliefs but also shaped by psychosocial factors and individuals’ stage of readiness to act.

### Ethical Considerations

The study was reviewed and approved by the institutional review board of the University of Minnesota (institutional review board protocol: STUDY00020835). All study procedures followed the ethical principles outlined in the Belmont Report and adhered to relevant federal and institutional guidelines for research involving human participants. Participants were informed about the purposes of the study, procedures, potential risks, and benefits through an online consent form prior to enrollment. Participants were reminded that their participation was voluntary, and they could withdraw from the study at any time without penalty. As the intervention and data collection took place online, additional measures were taken to ensure confidentiality, including secure data transmission, encrypted storage, and restricted access to identifiable information.

Minimal risks were anticipated, as the intervention involved completing online educational modules and surveys. The consent form explained potential discomfort that might arise from answering questions about smoking behavior and health beliefs. No adverse events were reported during the study, and no protocol deviation was noted from the study. Participants were compensated for their time with a US $40 Amazon gift card upon completion of the intervention, and an additional US $10 Amazon gift card was given to those who completed the 3-month follow-up survey.

All procedures complied with the Health Insurance Portability and Accountability Act as applicable, and the data were deidentified before analysis. Results are presented in an aggregate form to protect participants’ privacy.

### Setting

This study was conducted online. Interactive educational modules about LCS were created and implemented online through Research Electronic Data Capture (REDCap; Vanderbilt University). Questionnaires used in the study were administered on REDCap before and after the intervention. Follow-up emails were sent to increase the response rate of the questionnaires, and the study team answered any questions that the participants might have.

### Sample

The inclusion criteria for participants in this study were as follows: individuals with a smoking history of more than 20 pack-years, currently smoking or having quit smoking in the past 15 years, aged between 50 and 80 years, and able to read or understand English at the sixth-grade level. Exclusion criterion for the participants was having been diagnosed with lung cancer or other diseases that could substantially impact participants’ life expectancy. These criteria align with the US Preventive Services Task Force screening recommendation for lung cancer.

### Sample Size

The sample size was calculated using the G-Power software [21]. To detect a medium effect size (Cohen *d*=0.5) with an alpha error probability of .05 and a power of 0.95, a total sample size of more than 210 is required for a formal study when using a paired sample *t* test to detect differences. Considering data quality and the expected response rate of the questionnaires, an additional 20% increase in the suggested sample size is warranted, resulting in a target of 250 participants for the formal study.

As this study is a pilot study for the larger formal study, extant literature recommends that a pilot study should include approximately 10% of the sample size projected for the main study [21,22]. In addition, researchers typically suggest a sample size of 10 to 30 participants in both survey research and medical field research [23,24]. Therefore, a sample size of 25 participants would be appropriate for this pilot study.

### Recruitment Procedure

Webpages were created on REDCap to provide information about the study. Collaborating with the University of Minnesota Clinical and Translational Science Institute, all available social media platforms, including the StudyFinder (University of Minnesota) website, Instagram (Meta Platforms, Inc), and Facebook (Meta Platforms, Inc) groups, were used to recruit the planned number of participants. The study flyer was shared on these social media along with the research team’s email address. Once potential participants emailed the study team, they were screened for eligibility by answering 7 screening questions. If participants met the eligibility criteria, they were assigned a code number, and their contact information (eg, email address and cell number) was collected.

### Interventions

#### Preintervention Phase

Eligible participants were provided with a REDCap link for the questionnaires. Participants first read information about the study, reviewed the informed consent form, and then completed all preintervention questionnaires.

#### Intervention Phase

Participants who completed preintervention questionnaires were asked to review 5 online interactive modules on REDCap (including videos, text, pictures, and interactive activities) about lung cancer and its screening (Table 1).

**Table 1.** Lung cancer screening (LCS) online educational modules.

Module	Content
Module 1	Lung cancer epidemiology, etiology, signs, and symptoms
Module 2	Lung cancer treatment and care
Module 3	Lung cancer prevention methods
Module 4	LCS guidelines, benefits, and risks
Module 5	LCS procedures and results interpretation

#### Postintervention Phase

After reviewing the online educational modules, participants were asked to complete the postintervention questionnaires on REDCap. Participants were followed over 3 months [25] by emails to assess whether they underwent LCS.

### Development of Interactive Educational Modules

The interactive educational modules were developed through a 3-phase, user-centered process. First, a needs assessment was conducted through a literature review and individual qualitative interviews with 12 individuals with a heavy smoking history to identify knowledge gaps, misconceptions, preferred learning formats, and appropriate health literacy level. Second, 5 sequential modules were created featuring 3- to 5-minute artificial intelligence–generated video lectures, infographics, interactive quizzes, and reflection questions, with iterative prototyping to refine clarity and engagement and to align content with the HBM and PAPM constructs. Third, a panel of 7 clinical and behavioral experts evaluated accuracy and relevance; revisions were made based on item-level and scale-level Content Validity Index (CVI) results until strong content validity (item-level CVI≥0.78; scale-level CVI≥0.90) was achieved [26]. While this paper reported how the intervention performed during pilot testing, detailed methods for the design and development of these modules have been published elsewhere [27].

### Instruments

#### Sociodemographic Information

Sociodemographic characteristics were measured using the *Sociodemographic Information Questionnaire*, which included 8 questions on participants’ age, sex, marital status, level of education, annual family income, race, occupation, and health insurance status.

#### Intentions, Perceived Importance, and Confidence to Obtain LCS

Participants’ intentions, perceived importance, and confidence in undergoing LCS were assessed using *the Smoking and Lung Cancer Screening Questionnaire*, which was developed by the study team.

#### Knowledge of Lung Cancer and Screening

Participants’ knowledge of lung cancer and screening was assessed using 12 questions from the *Lung Cancer and Screening Knowledge Questionnaire* [28]. The questionnaire’s total score ranged from 0 to 12.

#### Attitudes Toward Lung Cancer Stigma

Nine questions about lung cancer stigma were assessed using the shortened version of the *Cataldo Lung Cancer Stigma Scale* [29], which uses a 4-point Likert-style response format. The scale’s total score ranged from 9 to 36 [30]. The reliability coefficient scores of the scale ranged from 0.75 to 0.96, which were in acceptable to excellent ranges [30].

#### Health Beliefs of Lung Cancer and Screening

Participants’ health beliefs regarding lung cancer and its screening were assessed using *the modified LCS Health Belief Scale*. This scale is composed of 57 items grouped into 6 subscales. The subscales for perceived severity, perceived susceptibility, perceived benefits, perceived barriers, and cues to action used 4-point Likert-style responses, with options ranging from strongly disagree (score=1) to strongly agree (score=4). The self-efficacy subscale also used 4-point Likert-style responses with options ranging from not at all confident (score=1) to very confident (score=4). This scale was developed from our preliminary studies, adopting the original *LCS Health Belief Scale* [31], which demonstrated acceptable content validity [32].

#### LCS Uptake

LCS uptake was assessed during the follow-up period after participants completed the online intervention. Upon enrollment, participants were informed that they would receive a follow-up email approximately 3 months later. This email inquired whether they had completed an LDCT LCS since participating in the study. Participants were instructed to respond with “yes” if they had undergone the screening and “no” if they had not. Only participants who replied to the follow-up email were included in the analysis.

#### Satisfaction Feedbacks

Participants’ feedback on the intervention was collected using the *Satisfaction Feedback Questionnaire* developed by the study team. The questionnaire consisted of 5 items, each measured using a 4-point Likert-style ordinal response scale. The total score for the questionnaire ranged from 5 to 20, with higher scores indicating greater satisfaction with the intervention. A total score above 15 indicated a positive attitude toward the online education modules.

### Data Analysis

Data analysis was done using SPSS (version 31.0.0; IBM Corp). Descriptive statistics, including mean and SD, frequency, and percentage, were used to evaluate the characteristics of the participants, participant satisfaction with the intervention, and LCS uptake rates. A paired *t* test was performed to examine differences in the mean scores of participants’ behavioral intention, perceived importance, and confidence toward LCS; knowledge of lung cancer and its screening; attitudes toward lung cancer stigma; and health beliefs regarding LCS before and after the intervention. The *P* value was set at .05. Results with a *P* value of <.05 were considered significant. Cohen *d* was calculated for paired comparisons to estimate the magnitude of change and inform future sample size calculations. The distributions of predifference and postdifference scores were examined using skewness, kurtosis, and Q-Q plots. Sensitivity analyses were conducted using nonparametric alternatives (Wilcoxon signed-rank tests), and results were compared for consistency.

## Results

### Participant Characteristics

A total of 27 participants were initially enrolled in the pilot study, of which 25 completed the intervention, resulting in a retention rate of 92.59%. The mean age of the participants was 60.88 (SD 5.16) years. Most participants were female (n=18, 72%), married (n=18, 72%), and had at least a bachelor’s degree (n=19, 76%). The majority reported a household annual income between US $45,000 and US $139,999 (n=19, 76%). Of the 25 participants, 44% (n=11) identified as White, 40% (n=10) as Black or African American, and 16% (n=4) as Hispanic or Latinx. More than half of the participants (n=14, 56%) were not currently employed, and 36% (n=9) did not have health insurance. The majority smoked currently (n=22, 88%), and none had previously undergone LCS. Only 1 (4%) participant reported a family history of lung cancer (Table 2). Average time for the participants completing the modules was 2 (SD 0.23) hours. All the participants completed the modules in a single setting.

**Table 2.** Participants’ characteristics (N=25).

Variables	Values
Age (years), mean (SD)	60.88 (5.16)
Sex, n (%)	
Female	18 (72)
Male	7 (28)
Marital status, n (%)	
Married	18 (72)
Separated	2 (8)
Divorced	2 (8)
Widowed	3 (12)
Education level, n (%)	
Some college	6 (24)
Bachelor’s degree	13 (52)
Graduate degree	6 (24)
Household annual income (US $), n (%)	
<20,000	1 (4)
20,000-44,999	2 (8)
45,000-139,999	19 (76)
140,000-149,999	3 (12)
Ethnicity, n (%)	
Hispanic or Latino	4 (16)
Not Hispanic or Latinx	21 (84)
Race, n (%)	
American Indian or Alaska Native	2 (8)
Asian	2 (8)
Black or African American	10 (40)
White	11 (44)
Currently employed, n (%)	
Yes	11 (44)
No	14 (56)
Insurance status, n (%)	
Have no insurance	9 (36)
Private insurance	8 (32)
Government insurance (Medicaid or Medicare)	7 (28)
Employee insurance	1 (4)
Smoking status, n (%)	
Individual who smokes currently	22 (88)
Individual who smoked formerly	3 (12)
Family history with lung cancer, n (%)	
Yes	1 (4)
No	24 (96)
LCS^a^ history, n (%)	
Yes	0 (0)
No	25 (100)

a LCS: lung cancer screening.

### Changes in LCS Intentions, Perceived Importance, and Confidence

Before the intervention, most participants (18/25, 72%) indicated that they had no plan to undergo LCS, while only 8% (2/25) intended to have the screening done within 6 months. However, after completing the online modules, their intentions to undergo screening increased substantially, with 52% (13/25) of the participants indicating plans to undergo screening within the next 6 months. Only 12% (3/25) of them stated that they did not plan to pursue it. Additionally, the perceived importance of LCS rose significantly (*t*_24_=−6.70; *P*<.001; Cohen *d*=−1.34). Confidence in the decision to undergo screening also improved (*t*_24_=−10.00; *P*<.001; Cohen *d*=−2.00; Table 3).

**Table 3.** Changes in lung cancer screening (LCS) intention, perceived importance, and confidence (N=25).

	Before the intervention	After the intervention
Are you planning to receive LCS with low-dose computed tomography later?, n (%)		
Within the next month	0 (0)	6 (24)
Within the next 2‐6 months	2 (8)	7 (28)
Sometime after 6 months	5 (20)	9 (36)
I am not planning to receive it	18 (72)	3 (12)
On a scale of 1-10, with 1=not at all important and 10=extremely important, how important is it for you to receive LCS with low-dose computed tomography?, mean (SD)	4.32 (2.27)	7.76 (1.72)
On a scale of 1-10, with 1=not at all confident and 10=extremely confident, how confident are you to receive LCS with low-dose computed tomography?, mean (SD)	4.12 (1.83)	7.84 (1.55)

### Changes in Lung Cancer Knowledge, Stigma, and Health Beliefs

Consistent with the theoretical framework, participants demonstrated significant improvement in their knowledge of lung cancer and its screening, perceived less stigma, and developed more positive health beliefs about LCS. The average knowledge scores increased from 3.76 (SD 2.26) to 8.60 (SD 2.27; *t*_24_=−7.28; *P*<.001; Cohen *d*=−1.46). Notably, lung cancer stigma decreased from 25.52 (SD 4.72) to 19.16 (SD 4.62; *t*_24_=6.28; *P*<.001; Cohen *d*=1.26). Changes were observed across all subscales of the modified LCS Health Belief Scale: perceived susceptibility (mean 8.2, SD 2.06 vs mean 10.44, SD 2.16; *t*_24_=−4.63; Cohen *d*=−0.93), benefits (mean 17.72, SD 3.09 vs mean 24.12, SD 3.84; *t*_24_=−7.51; Cohen *d*=−1.5), cues to action (mean 15.28, SD 3.29 vs mean 20.84, SD 3.30; *t*_24_=−8.08; Cohen *d*=−1.62), and self-efficacy (mean 28.36, SD 4.89 vs mean 35.28, SD 4.45; *t*_24_=−5.79; Cohen *d*=−1.16). Meanwhile, perceived barriers decreased (mean 55.64, SD 5.50 vs mean 44.24, SD 6.95; *t*_24_=9.09; Cohen *d*=1.82) and severity dropped (mean 26.72, SD 4.87 vs mean 21.76, SD 2.86; *t*_24_=4.00; Cohen *d*=0.80). All changes were statistically significant (all *P* values <.001).

The Cohen *d* test showed large effect sizes (Cohen *d* >0.80) for all outcomes. Inspection of the distributions of predifference and postdifference scores suggested no substantial deviations from normality. The results of the Wilcoxon signed-rank tests were consistent with the paired *t* test findings, with no meaningful differences in statistical significance or direction of effects (*P*<.01).

### LCS Uptake

Of the 22 participants who replied to the follow-up email, 13 (59.1%) reported that they had completed LCS with LDCT, while 9 (40.9%) reported not having undergone the screening. The attrition rate for the follow-up was 12% (3/25). The self-reported rate indicated that more than half of those who responded engaged in screening following the online intervention.

### Satisfaction Toward the Intervention

Overall, participants reported high satisfaction with the online education intervention program. On a 4-point Likert scale, the average satisfaction score was 18.32 (SD 2.33). All participants described the online teaching session as informative and helpful, noting that their interest and motivation in learning increased afterward. In total, 96% (24/25) of the participants reflected that they had learned and understood the materials presented during the session; they felt that the session covered all the stated objectives.

## Discussion

### Principal Findings

This pilot study demonstrated that an online, theory-based educational intervention can effectively improve knowledge, attitude, beliefs, behavioral intentions, perceived importance, and confidence of high-risk individuals regarding LCS. Following the intervention, participants reported significant increases in the intention, perceived importance, and confidence in obtaining an LDCT screening. There was an increase in knowledge about lung cancer and screening, a decrease in perceived stigma toward lung cancer, improved health beliefs regarding LCS, and a high level of satisfaction with the program. Compared with existing digital LCS decision aids such as LungTalk and mPATH-Lung, this study extends prior work by focusing on a fully remote, multisession educational approach aimed at modifying health beliefs and supporting progression toward screening intention outside of clinical encounters.

The observed improvements align with previous studies, demonstrating that targeted educational interventions can effectively modify cognitive and affective determinants of screening behavior [14,16-18]. In addition, this study contributes to existing literature by delivering the intervention entirely online, thus overcoming geographic and logistical barriers often encountered with traditional face-to-face formats.

The notable increases in the intentions, perceived importance, and confidence regarding undergoing LCS indicate that the intervention effectively influenced participants’ readiness for behavior change. This finding suggests that digital delivery can achieve similar behavioral impacts while enhancing accessibility. According to the HBM and PAPM, importance reflects perceived value and outcome expectancy, while confidence, often conceptualized as self-efficacy, is a key predictor of behavioral intentions to act [33]. The simultaneous rise in both constructs suggests that the online modules not only provided information but also effectively addressed the motivational and self-regulatory components for behavior change.

The substantial increase in knowledge about lung cancer and improvements across all areas of the Health Belief Scale, such as susceptibility, benefits, barriers, cues to action, and self-efficacy, demonstrate that the intervention engaged multiple cognitive pathways that influence health behavior. Participants not only acquired new information but also internalized their personal risk. They began to view screening as beneficial, perceived fewer barriers, and reported stronger cues to action. These comprehensive changes suggest that the educational modules served as a multifaceted behavioral intervention rather than a simple knowledge-based program.

One unexpected finding was the observed decrease in perceived severity following the intervention. In the context of LCS, perceived severity is often uniformly high at baseline, as lung cancer is widely recognized as a serious and life-threatening condition, and prior research has noted potential ceiling effects [31]. The decrease observed in this study may reflect improved knowledge and reduced fatalistic beliefs rather than a diminished understanding of disease seriousness. Specifically, after the educational intervention, participants may have developed a clearer understanding of available treatment options and the benefits of early detection, including the substantially higher survival rates associated with early-stage lung cancer. This shift may have led participants to perceive lung cancer as more treatable when detected early, thereby lowering perceived severity scores.

Importantly, this interpretation suggests a cognitive reframing from viewing lung cancer as uniformly fatal to recognizing it as a condition with meaningful opportunities for early intervention and improved outcomes. However, consistent with LCS-specific conceptual models, reducing perceived severity alone is unlikely to directly motivate screening behavior. Instead, constructs such as perceived risk (susceptibility), perceived benefits, and self-efficacy, each of which improved in this study, are more proximal determinants of screening uptake. Future studies should prioritize these constructs and consider refining measurement approaches to better align with validated LCS-specific frameworks.

Furthermore, the marked reduction in stigma surrounding lung cancer is particularly noteworthy, which supports earlier findings that education and normalization of LCS can mitigate blame and fatalistic attitudes among individuals who smoke [34]. Stigma has long been recognized as a psychological barrier that discourages individuals who smoke from participating in LCS due to feelings of shame, blame, or fear of judgment. By presenting lung cancer as a preventable and treatable condition if detected early and by portraying screening as a responsible health decision rather than a consequence of smoking, the intervention may have helped shift participants’ views of lung cancer from self-blame to empowerment. This change is crucial because lower stigma is linked to higher screening uptake and better engagement with health care services [34].

The reported uptake rate (13/25, 59%) of LCS is substantially higher than national estimates of LCS participation, which are approximately 18% among eligible individuals in the United States [35]. Although a higher LCS rate is observed in this study, it should not be interpreted as a direct indicator of intervention effectiveness. The discrepancy may reflect differences in sample characteristics, such as higher motivation among study participants, as well as potential response and reporting biases.

Participants expressed exceptionally high satisfaction with the online educational program. Satisfaction is a crucial yet often overlooked component in digital health interventions, as it is closely linked to engagement, adherence, and long-term retention. The consistently high ratings across all satisfaction indicators suggest that the format, content, and delivery were well aligned with adult learning principles and the needs of the target population. High satisfaction also increases the likelihood that participants may recommend the program to their peers, which could extend the intervention’s reach through community engagement.

### Strengths

This study introduces a novel, theory-based online educational intervention grounded in the HBM and PAPM. Unlike prior approaches, this intervention was designed as a multicomponent, interactive program that integrates short video-based learning, quizzes, and reflective activities to actively engage users and reinforce key behavioral constructs, including perceived susceptibility, perceived benefits, and self-efficacy. Additionally, the intervention was developed using a user-centered design process informed by qualitative data from high-risk individuals, ensuring that content is culturally and contextually relevant, accessible, and aligned with participants’ health literacy levels.

Another innovative aspect of this study lies in its emphasis on addressing lung cancer–related stigma and psychological barriers, which are often undertargeted in existing interventions. By incorporating content that normalizes screening, reduces blame, and reframes lung cancer as a preventable and treatable condition when detected early, the intervention extends beyond knowledge enhancement to target affective and social determinants of health behavior.

### Limitations and Future Directions

This study has notable limitations due to its small sample size and single-arm preintervention and postintervention design, which affects its generalizability and the ability to infer causal relationships. Without a control or comparison group, it is not possible to determine whether the observed changes were attributable to the intervention itself or alternative explanations, such as repeated measurement effects, regression to the mean, social desirability bias, or natural changes over time. Therefore, the findings should be interpreted as preliminary and hypothesis generating. Future research using randomized controlled designs with larger samples and comparison groups reflective of standard clinical practice (eg, clinical counseling with primary care providers) is needed to establish the efficacy of the intervention.

In addition, the generalizability of the findings is limited by the sociodemographic characteristics of the sample. Participants in this study were relatively well educated and reported moderate to higher income levels, which may not reflect the broader population eligible for LCS, who often have lower educational attainment and fewer financial resources. Furthermore, over one-third of participants reported lacking health insurance, highlighting a critical structural barrier to LCS uptake. Future research should prioritize the development and evaluation of interventions that are accessible to individuals with lower health literacy, and integrating educational interventions with system-level supports, such as patient navigation, insurance assistance, and linkage to affordable screening services, may be necessary to effectively reduce disparities in LCS uptake.

Finally, at the 3-month follow-up, LCS uptake was assessed via participant self-report collected through email. However, this measure was not verified through medical records or imaging data and should therefore be interpreted with caution. In addition, 3 participants did not respond to the follow-up assessment and were not included in this estimate. No comparison between responders and nonresponders was conducted. These missing data may further limit the validity and generalizability of the findings. Future research should use objective screening documentation to confirm whether participants completed LCS and conduct qualitative studies to identify specific motivational or structural factors that influence screening uptake.

### Conclusions

This pilot study yielded promising findings, suggesting that an online, interactive educational intervention grounded in the HBM and PAPM can enhance knowledge, attitude, beliefs, and readiness for LCS among high-risk individuals who smoke. The program was well received, user-friendly, and showed measurable psychosocial benefits. While promising, these findings require confirmation in rigorously designed controlled trials before conclusions about effectiveness can be drawn. With further testing and refinement, this intervention has the potential to serve as a scalable public health tool aimed at reducing disparities in lung cancer through extensive reach, improved prevention, and early detection.

## Abbreviations

CVI: Content Validity Index
HBM: health belief model
LCS: lung cancer screening
LDCT: low-dose computed tomography
PAPM: precaution adoption process model
